# Tenodesis of the Iliotibial Tract in the Treatment of Lesions of the Anterolateral Knee Complex – Description of a Modified Technique

**DOI:** 10.1055/s-0045-1811630

**Published:** 2025-11-10

**Authors:** Gustavo Rabelo Azi, Alexandre Vasconcelos de Meirelles, Ramon Rocha Costa de Freitas, José Fonseca Fróes Neto, Enilton de Santana Ribeiro de Mattos, Alex Guedes

**Affiliations:** 1 Knee Surgery Group, Hospital Santo Antônio, Obras Sociais Irmã Dulce, Salvador, BA, Brazil; 2 Medical Residency Program in Orthopedics and Traumatology, Hospital Santo Antônio, Obras Sociais Irmã Dulce, Salvador, BA, Brazil; 3 Hip Surgery Group, Hospital Santo Antônio, Obras Sociais Irmã Dulce, Salvador, BA, Brazil; 4 Hand Surgery Group, Hospital Santo Antônio, Obras Sociais Irmã Dulce, Salvador, BA, Brazil; 5 Knee Surgery Group, Hospital Universitário Professor Edgard Santos, Faculdade de Medicina da Bahia, Universidade Federal da Bahia, Salvador, BA, Brazil

**Keywords:** anterior cruciate ligament reconstruction, fascia lata, joint instability, knee, ligaments, operative surgical procedures, fáscia lata, instabilidade articular, joelho, ligamentos, procedimentos cirúrgicos operatórios, reconstrução do ligamento cruzado anterior

## Abstract

The authors describe a modified lateral extraarticular tenodesis technique for the treatment of anterolateral complex knee injuries, performed in association with anterior cruciate ligament reconstruction, using a quadruple semitendinosus and gracilis tendon graft. Femoral fixation of the graft is performed with a single interference screw, together with the central band of the iliotibial tract, from the outside in, sharing the same bone tunnel, whose entrance is positioned in the topography of the lateral epicondyle (at the level of the origin of the anterolateral ligament). The postoperative protocol includes physiotherapy during the first 4 months, followed by the initiation of muscle strengthening, with clearance for return to sports starting from the 9 ^th^ month.

## Resumo

Os autores descrevem uma técnica modificada de tenodese extrarticular lateral para o tratamento das lesões do complexo anterolateral do joelho, realizada em associação à reconstrução do ligamento cruzado anterior, utilizando enxerto tendíneo quádruplo de semitendíneo e grácil. A fixação femoral do enxerto é realizada com um único parafuso de interferência, de fora para dentro, compartilhando o mesmo túnel ósseo com a banda central do trato iliotibial. A entrada do túnel é posicionada na topografia do epicôndilo lateral, ao nível da origem do ligamento anterolateral. O protocolo pós-operatório inclui fisioterapia nos primeiros 4 meses, seguida pelo início do reforço muscular, com liberação para a prática esportiva a partir do 9° mês.

## Introduction

Anterolateral rotatory instability (ARI) results from anterior cruciate ligament (ACL) rupture and injury to the anterolateral complex of the knee (a portion of the iliotibial tract [ITT] located between the Kaplan fibers proximally and its tibial attachment), 1 2 generating anomalous tibial anterior translational and rotational (internal) movement. 2

Failure to recognize and manage concomitant injuries of the anterolateral knee complex at the time of ACL reconstruction may result in persistent ARI, culminating in poor functional outcomes, reconstruction failure, neoligament rupture, and progressive osteoarthritis. 2 3 As such, the concomitant surgical approach to the anterolateral complex, including the anterolateral ligament (ALL) and the iliotibial band (ITB), has become the object of increasing interest and investigation, as it allows the re-establishment of rotational stability, restoring normal knee biomechanics, reducing failure rates of isolated ligament reconstruction, and preventing or minimizing other complications. 2 3 4 5 6

Currently, anterolateral instability treatment uses two techniques: lateral extraarticular tenodesis (LET) 3 7 8 9 and ITT reconstruction. 2 4

Lateral extraarticular tenodesis 3 7 8 9 involves passing the ITT through a tunnel in the femur followed by its fixation to this bone, providing knee rotational containment and lateral stabilization.

The present study aims to describe a modified LET technique for the concomitant treatment of combined ACL and anterolateral knee complex injuries in reconstructions using a quadruple semitendinosus and gracilis tendon graft. Femoral graft and ITT central band fixation occurs at the same time, using the same bone tunnel.

## Technique Description


**Video 1**


We place the patient in the supine position. Anesthesia involves spinal block and sedation. We complete the prophylactic intravenous antibiotic therapy up to 30 minutes before the incision using 1 g of cefazolin or 600 mg of clindamycin (for patients allergic to beta-lactam antibiotics).

**Fig. 1:**
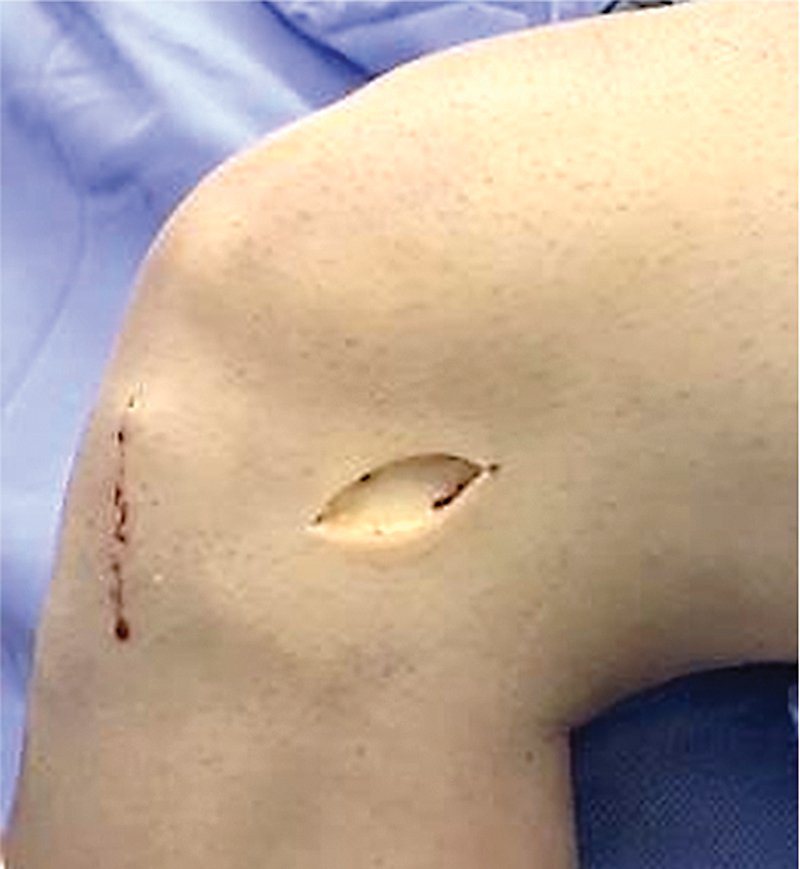
Mini-approach over the iliotibial tract.

We perform venous emptying by gravitational elevation of the affected limb for 5 minutes. Next, we use an Esmarch bandage and a proximal tourniquet at the root of the thigh with a pneumatic cuff. With the knee flexed at 90° and the leg hanging next to the operating table, we perform the routine approach for anatomical ACL reconstruction using the quadruple semitendinosus and gracilis tendon graft (prepared by suturing the ends and folding the graft in half its length, leaving the suture thread around the fold), except for creating a femoral tunnel from the outside to the inside, with its entrance at the ALL origin, 0.5 cm proximal and 0.5 cm posterior to the lateral epicondyle of the femur. To do so, we perform a mini cutaneous approach (∼ 2.0 cm) in the topography of the lateral epicondyle, proceeding with dissection by planes, identification and dissection of the ITT ( Fig. 1 ). After dissection, we incise the ITT longitudinally (from 5.0 to 7.5 cm), following the orientation of its fibers; the separation of the edges of this incision allows the identification of the lateral epicondyle of the femur ( Video 1 ).

Next, we position a drill-tip guidewire 0.5 cm proximal and 0.5 cm posterior to the lateral epicondyle of the femur, directing it to the center of the ACL footprint with a pinpoint guide; then, we create a femoral tunnel from the outside to the inside. We calculate the femoral tunnel diameter for graft passage. We prepare a tibial tunnel from the outside to the inside, positioning the guide in the footprint or over the remnant of the ACL in the tibia to ensure an anatomical reconstruction ( Video 1 ).

**Fig. 2:**
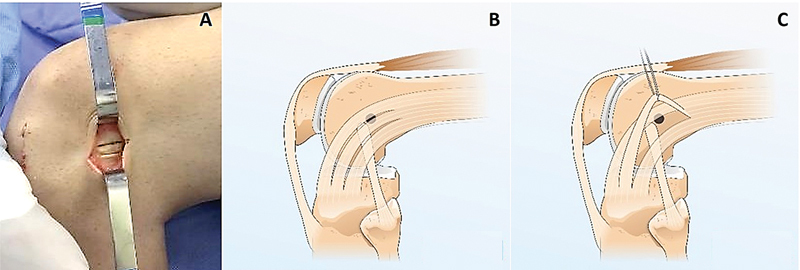
( **A-C** ). ( **A** ) Lateral aspect of a left knee showing the incision in the iliotibial tract; ( **B** ) Illustration of the iliotibial tract graft and the point of femoral tunnel creation; ( **C** ) pulling the iliotibial tract graft with suture.

**Fig. 3:**
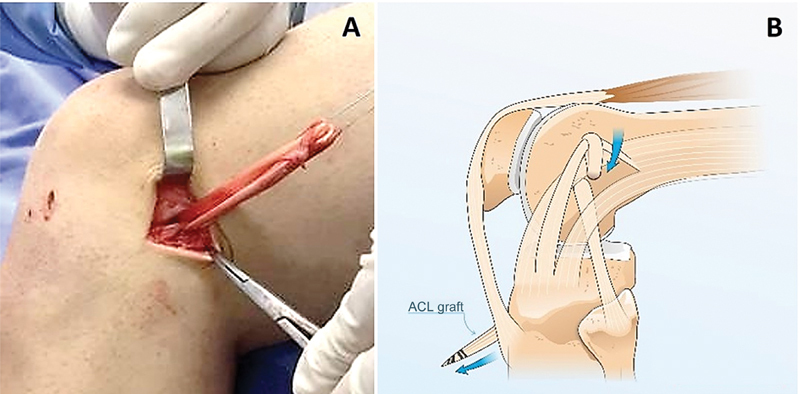
( **A-B** ). ( **A** ) Graft from the central band of the iliotibial tract; ( **B** ) Passage around the flexor graft. ACL, Anterior cruciate ligament.

**Fig. 4:**
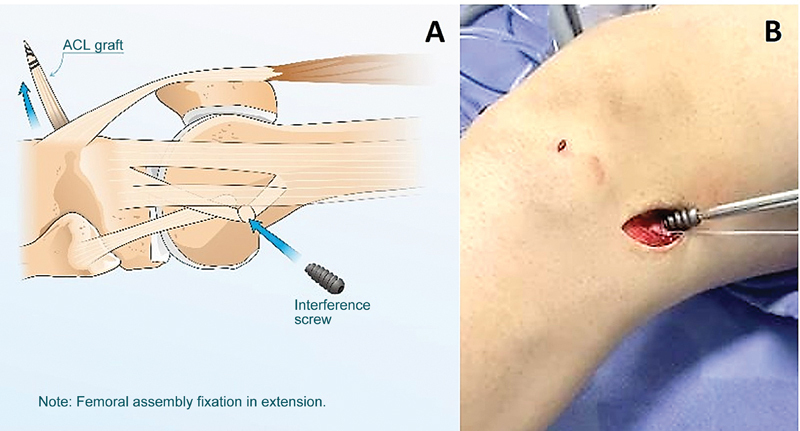
( **A-B** ). Central band of the iliotibial tract and quadruple semitendinosus and gracilis graft fixation in the femoral tunnel using a screw with the knee in extension. ACL, Anterior cruciate ligament.

We perform a new incision in the ITT, parallel to the initial one but 0.5 cm away from it, creating a central band measuring 5.0 to 7.5 × 0.5 cm, maintaining continuity with its proximal and distal portions ( Fig. 2 ). We position the graft from proximal to distal around the band ( Fig. 3 ) and pull it to the joint using high-resistance thread (Ethibond 5, Johnson & Johnson) for a few millimeters, inside the femoral tunnel; then, with the knee in neutral rotation and in full extension, we fix these structures with an interference screw ( Fig. 4 , Video 1 ).

**Fig. 5:**
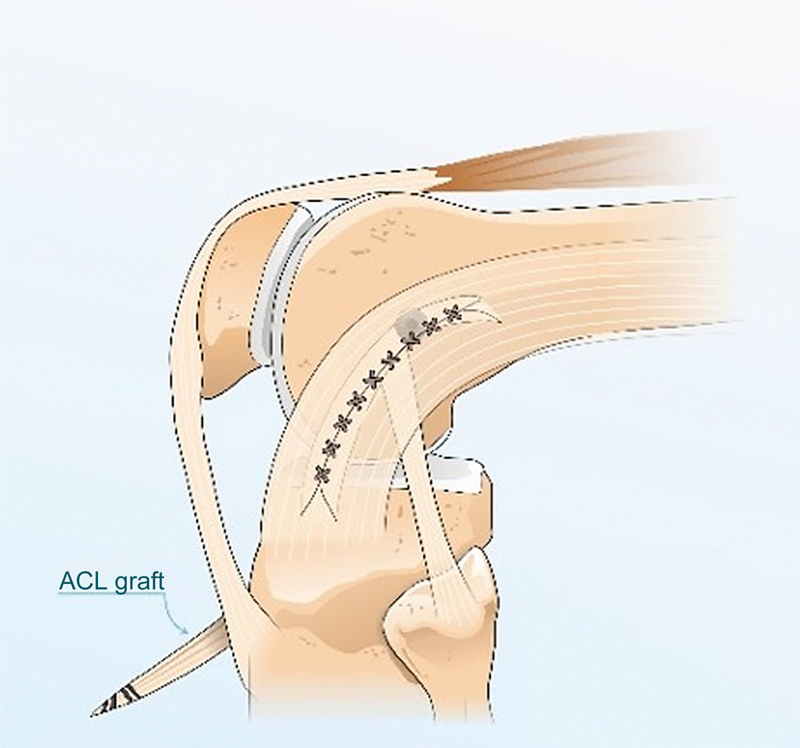
Suture of the central band of the iliotibial tract. ACL, Anterior cruciate ligament.

Next, with the knee at 30° of flexion, we fix the graft in the tibial tunnel with an interference screw. We suture the gap from the invagination of the central band of the ITT in the femoral tunnel with the knee in flexion ( Fig. 5 , Video 1 ) using interrupted X-shaped stitches of Vycril 0 (Ethicon or Johnson & Johnson Medical Devices). We complete the procedure by approximating the wound in layers using Vycril 2–0 (subcutaneous tissue, simple interrupted suture with inverted knot) and Mononylon 3–0 (Ethicon or Johnson & Johnson Medical Devices) (skin, separate simple stitches), compressive dressing, and conventional radiographic follow-up examination. We do not use aspiration drains or knee immobilizers.

The patient remains hospitalized for 1 day, and we allow them to bear full weight on the 1 ^st^ postoperative day, as tolerated, using crutches only for balance. Venous thromboembolism prophylaxis is exclusively mechanical, through intermittent mechanical compression, elastic stockings, or both. We perform local cryotherapy and, as required, pain control with non-opioid analgesics. Suture removal occurs after 15 days.

Follow-up occurs once a month until resuming sports activities 9 months after surgery. Postoperative rehabilitation consists of the following protocol:

- From the immediate postoperative period to the third month after surgery: physical therapy for analgesia, gain of joint range of motion , and prevention of muscle mass loss .
- From the third to the sixth month after surgery: gradual transition from physical therapy to physical exercise, focusing on progressive muscle mass gain.
- From the third to the ninth month after surgery: neuromuscular and sports gesture training under the supervision of a physical educator.
- Nine months after surgery: clinical evaluation for sports practice clearance, including muscle mass analysis, ligament tests, hop test, and follow-up magnetic resonance imaging scans.

## Final Comments

Anterolateral rotatory instability results from ACL rupture and injury to the anterolateral complex of the knee. The identification of this condition requires planning a concomitant surgical approach, associating ACL reconstruction with LET 3 7 8 9 or ITT reconstruction. 2 4 Primary indications for this procedure include ACL re-rupture, pivot-shift grade 2 or 3 at physical examination, sports practice with pivot movements and/or high-level sports practice, ligamentous laxity, and Segond fracture; secondary indications include chronic ACL injury, age < 25 years old, and radiographic notch sign on the lateral femoral condyle. 10

Lateral extraarticular tenodesis is a widely studied technique 3 7 8 9 introduced by French surgeon Marcel Lemaire in 1967. 3 As its precise role and ideal technique are controversial, several proposed modifications have emerged as preferred due to their significant effectiveness in reducing failure rates in isolated ACL reconstructions. 3 7 8 9

Despite the benefits, it is crucial to consider that LET has some potential for complications, 8 including pain, restriction of joint range of motion (especially flexion, due to tensioning of the ITT), fixation failure and graft rupture.

In the modified TEL, described in the present study, the femoral tunnel created for passing the quadruple semitendinosus and gracilis tendon graft also receives the central band of the ITT. The graft and the band undergo fixation together with a single interference screw. In this technique, the point defined for creating the femoral tunnel (from the outside to the inside, 0.5 cm proximal and 0.5 cm posterior to the lateral epicondyle of the femur, directed toward the center of the ACL footprint) considers performing the tenodesis at the site closest to the origin of the anterolateral ligament, without compromising the positioning of the quadruple tendon graft.

This procedure has the following benefits: (1) single mini-approach, with no need for a new incision for LET; (2) technically simpler procedure; (3) shorter surgical time (lower tourniquet time); (4) no requirement to modify the postoperative rehabilitation protocol for ACL reconstruction due to LET; (5) lower implant demand; and (6) lower cost resulting from the previous items.

The potential risk for complications is consistent with other LET techniques, including (1) pain; (2) flexion restriction; (3) fixation failure; and (4) graft rupture.
